# BMS794833 inhibits macrophage efferocytosis by directly binding to MERTK and inhibiting its activity

**DOI:** 10.1038/s12276-022-00840-x

**Published:** 2022-09-02

**Authors:** Seung-Hyun Bae, Jung-Hoon Kim, Tae Hyun Park, Kyeong Lee, Byung Il Lee, Hyonchol Jang

**Affiliations:** 1 Reasearch Institute, National Cancer Center, Goyang, 10408 Republic of Korea; 2 Department of Cancer Biomedical Science, National Cancer Center Graduate School of Cancer Science and Policy, Goyang, 10408 Republic of Korea; 3 Department of Anesthesiology, Weill Cornell Medical College, New York, NY 10065 USA; 4 College of Pharmacy, Dongguk University-Seoul, Goyang, 10326 Republic of Korea

**Keywords:** Biochemistry, Cell biology

## Abstract

Myeloid epithelial reproductive proto-oncogene tyrosine kinase (MERTK) plays an essential role in modulating cancer immune tolerance by regulating macrophage efferocytosis. Studies are underway to develop small-molecule chemicals that inhibit MERTK as cancer immunotherapeutic agents, but these efforts are in their early stages. This study identified BMS794833, whose primary targets are MET and VEGFR2, as a potent MERTK inhibitor and developed a real-time efferocytosis monitoring system. The X-ray cocrystal structure revealed that BMS794833 was in contact with the ATP-binding pocket and the allosteric back pocket, rendering MERTK inactive. Homogeneous time-resolved fluorescence kinetic and Western blotting analyses showed that BMS794833 competitively inhibited MERTK activity in vitro and inhibited the autophosphorylation of MERTK in macrophages. We developed a system to monitor MERTK-dependent efferocytosis in real time, and using this system, we confirmed that BMS794833 significantly inhibited the efferocytosis of differentiated macrophages. Finally, BMS794833 significantly inhibited efferocytosis in vivo in a mouse model. These data show that BMS794833 is a type II MERTK inhibitor that regulates macrophage efferocytosis. In addition, the real-time efferocytosis monitoring technology developed in this study has great potential for future applications.

## Cancer: drug could block evading the immune system

An enzyme that is over-expressed in cancers and helps promote immune evasion could be inhibited by a small-molecule anticancer drug currently in phase 1 clinical trials. White blood cells called macrophages rapidly clear dead cells to avoid inappropriate inflammatory responses in a process known as efferocytosis. The MERTK enzyme receptor, expressed by macrophages, plays a key role. However, MERTK is over-expressed in many cancers, leading to aberrant efferocytosis and immune evasion. Using mouse models, Hyonchol Jang and Byung Il Lee at the National Cancer Center, Goyang, South Korea, and co-workers demonstrated that the drug BMS794833 is an effective inhibitor of MERTK-dependent efferocytosis. Using X-ray crystallography, they determined that the drug binds to and inactivates MERTK. To potentially identify other MERTK inhibitors, they also developed a novel assay that monitors efferocytosis in real time.

## Introduction

Apoptotic cells are rapidly cleared via phagocytes to prevent inappropriate inflammatory responses and maintain physiological homeostasis in a process known as efferocytosis^1,2^. Efferocytosis is conducted by macrophages that express many receptors, including myeloid epithelial reproductive proto-oncogene tyrosine kinase (MERTK)^1^. MERTK is a member of the TAM (TYRO3, AXL, and MERTK) receptor tyrosine kinase family. These kinases play essential roles in immune homeostasis and are involved in regulating the innate immune response, restoring vascular integrity, and efferocytosis by different phagocytic cell types^3,4^. The role of MERTK in regulating efferocytosis is most prominent among the TAM family, considering that clearance of apoptotic thymocytes is reduced in *Mertk*^*–/*–^ mice but normal in *Axl/Tyro3*^*–/–*^ mice^4,5^. MERTK is activated by autophosphorylation when protein S or growth arrest-specific 6 (GAS6) ligands that bind to phosphatidylserine (PtdSer) are exposed on the surface of dying or dead cells bind to MERTK. Macrophages with activated MERTK begin to engulf apoptotic cells through actin reorganization^3^. They also engulf foreign antigens through phagocytosis, but whether MERTK participates in this process is unclear.

MERTK is aberrantly expressed in various cancers and promotes tumor immune evasion and M2 polarization of macrophages through the regulation of efferocytosis^6^. Studies on *Mertk*^*–/*–^ mice, small-molecule MERTK inhibitors, and MERTK-specific antibodies have demonstrated that MERTK inhibition has an anticancer effect and that regulation of efferocytosis is a key mechanism^6–8^. To develop MERTK inhibitors as anticancer drugs, it is necessary to confirm whether candidate substances inhibit efferocytosis by inhibiting MERTK. Recently, human monocytes were extracted and differentiated into macrophages to monitor the efferocytosis ability of macrophages in vitro^9^. However, it is time-consuming and difficult to obtain a sufficient number of monocytes. In addition, it is not known whether the decrease in the efferocytosis ability of macrophages is due to MERTK inhibition. Therefore, a system to easily monitor MERTK-dependent efferocytosis is needed.

Structural studies of eukaryotic protein kinase domains have reported characteristic conformational changes in the conserved Asp-Phe-Gly (DFG) motif and the αC-helix (αC) during enzymatic activation^10–12^. Therefore, the active/inactive state of kinases can be determined by DFG and αC conformation: the active conformation of kinases should adopt the “DFG flip-in” and “αC swing-in” conformation; all other conformations in DFG and αC are inactive kinases. ATP competitive protein kinase inhibitors can be classified into three types. Type I inhibitors bind to the ATP-binding pocket of the active conformation of kinases that have DFG-in and αC-in structures, type II kinase inhibitors bind to the inactive DFG-out conformation, and type I1/2 inhibitors bind to the inactive DFG-in conformation with an αC-out form^13^.

Some small molecules with MERTK inhibitory effects have been used clinically, but in all cases, MERTK is not their primary target, and in most cases, their binding mode with MERTK has not been clearly elucidated. Some small molecules that primarily target MERTK are in clinical trials, but in all cases, their binding mode with MERTK has not been clearly elucidated by X-ray crystallography. MERTK inhibitors with clearly defined binding modes, except for gilteritinib, AZD7762, and merestinib, have advanced at most to the preclinical stage. Moreover, the primary targets of gilteritinib, AZD7762, and merestinib are not MERTK but FLT3, CHEK1, and MET, respectively^14–16^. Thus, the development of anticancer drugs that specifically inhibit MERTK is still at an early stage.

In this study, we investigated the binding mode between MERTK and BMS794833 through X-ray crystallography. BMS794833 is a small molecule in phase I clinical trials with BMS817378, a prodrug of BMS794833 that targets advanced or metastatic solid tumors (ClinicalTrials.gov identifier: NCT00792558), in which the primary targets are MET and VEGFR2^17^. We also developed a system to monitor MERTK-dependent efferocytosis using monocyte cell line-derived macrophages in real time. BMS794833 inhibited MERTK activity in vitro and in cells and inhibited efferocytosis in cells and animals.

## Materials and methods

### Cloning and protein purification

Whole cloning and purification protocols followed a previous report^15^. In a biochemical activity assay, the gene coding the N-terminal His_6_ tag-thrombin-MERTK kinase domain (residues 571–864, wild type; WT) was subcloned into the pETDuet-1-PTPN1 (pETDuet-1 vector containing the PTPN1 (1–330) gene at MCS2, Merck Millipore, USA) bacterial expression vector using the Nco1 and Not1 cloning sites at MCS1. In addition, surface entropy reduction mutations were introduced (GSHM-MERTK K591R/K693R/K702R/K856R; KR mutant)^18^ via site-directed mutagenesis to improve the quality of the protein crystals.

### Crystallization and structure determination

All crystals were prepared via the sitting-drop vapor-diffusion method using Mosquito® (SPT Labtech, UK) at 14 °C. MERTK native crystals were obtained with 15 mg/mL purified recombinant MERTK (KR mutant) and a crystallization reservoir solution of 0.1 M Tris-HCl pH 8.5 and 4.3 M NaCl. Microseeding increased the crystal size and improved the diffraction quality. Native crystals were obtained within a day but grown fully for 5–7 days.

To obtain ligand-bound forms of MERTK, crystals were prepared by the seeding method. Freshly thawed MERTK:BMS794833 (25 mg/mL protein and 2 mM ligand) was incubated for 2 h under ice-cold conditions. Crystallization was achieved following the same protocol described above. Nine days after crystallization started, the crystals were transferred to a cryoprotectant solution (0.1 M Tris-HCl pH 8.5, 2.5 M NaCl, 20% DMSO, and 10 mM BMS794833) for X-ray diffraction experiments.

Diffraction data were collected using a Dectris Pilatus 6 M CCD detector at the beamline-11C experimental station at the Pohang Accelerator Laboratory (Korea). The structure was solved by molecular replacement with the PHASER program^19^ using the MERTK kinase domain structure (PDB code 7AAX^18^) as the search model. Repeated rounds of manual model building and crystallographic refinement were performed using COOT^20^ and PHENIX^21^. The topology and parameter files of the inhibitor were built by the Electronic Ligand Builder and Optimization Workbench (eLBOW) suite in PHENIX. The inhibitor was fitted to 2mF_o_-Df_c_ electron density, and further refinement cycling was repeated. The refinement statistics are summarized in Supplementary Table 1.

### HTRF-based MERTK kinetic assays

Kinetic assays for the MERTK kinase domain were conducted using a homogeneous time-resolved fluorescence (HTRF) KinEASE-TK kit (Cisbio, USA) as described previously^15^. Freshly thawed MERTK WT was serially diluted and used at 1 ng/μL (final) for the assay. We added 5 mM MgCl_2_, 1 mM DTT, and 0.2% DMSO to the reaction buffer. The enzymatic reaction mixture was incubated for 120 min at 37 °C. Time-resolved fluorescence intensities were recorded by TECAN SPARK with an excitation wavelength of 320 nm and two emission wavelengths of 620 and 650 nm. The integration and lag times were used as the default settings (400 and 100 μs for each). The IC_50_ values for the inhibitor were determined at 30 μM (close to *K*_m_) and 1 mM ATP (at the cellular level) with an inhibitor concentration of 0.000067–4 μM. Independent triplicate assay experiments confirmed the reproducibility of the assays.

### Cell cultures

All cell lines were purchased from the Korean Cell Line Bank (Seoul, Korea). THP-1, U-937, and Jurkat cells were cultured in RPMI-1640 medium (HyClone, USA) supplemented with 10% heat-inactivated fetal bovine serum (HyClone, USA), 1% penicillin/streptomycin (Invitrogen, USA), and 1× 2-mercaptoethanol (Gibco, USA) in T25 or T75 flasks at 37 °C in a humidified incubator containing 5% CO_2_. The cells were authenticated and checked for *mycoplasma* at the Genomics Core Facility (National Cancer Center, Korea) as described previously^22^.

### Real-time monitoring of efferocytosis and phagocytosis

A total of 50,000 THP-1 and U-937 cells were differentiated into macrophages by treatment with 100 nM phorbol-12-myristate 13-acetate (PMA; Sigma, USA) for 2 days in each well of a 96-well Cellcarrier Ultra microplate (Perkin Elmer, Germany). Simultaneously, to induce apoptosis, Jurkat cells were treated with 50 µM cisplatin (Sigma, USA) in a T75 flask for 2 days. Jurkat cells were stained with 50 ng/mL pHrodo™ Red and succinimidyl ester (pHrodo-SE; Thermo Fisher Scientific, USA) for 1 h, and then 200,000 cells/well were added to the macrophage culture wells. If necessary, inhibitors were used to treat macrophages 1 h before the addition of Jurkat cells. Images were obtained using Operetta CLS™ (Perkin Elmer, Germany) at a 1 h interval for 24 h and analyzed with Harmony 4.6 software (Perkin Elmer, Germany).

For the phagocytosis assay, differentiated macrophages were added to 20 μg of pHrodo™ Red *S*. *aureus* Bioparticles™ (pHrodo-particles; Thermo Fisher Scientific, USA) per well instead of stained Jurkat cells. The pHrodo™ Red dye is pH-sensitive; it is colorless at neutral pH but acquires red fluorescence (Ex/Em λ: 560/585 nm) in acidic environments of approximate pH 5–6. Macrophages swallowing pHrodo-SE-stained apoptotic cells or pHrodo-particles exhibit red fluorescence.

### Mice and in vivo efferocytosis assay

Female C57BL/6 N mice were purchased from Orient Bio (Seongnam, Korea). All mice were acclimatized for 2 weeks at the laboratory animal research facility (National Cancer Center, Korea). The mice were administered 25 mg/kg BMS794833 (Cayman, USA) or control (5% DMSO, 10% kolliphor in PBS) by intraperitoneal injection. After 30 min, 8 mg/kg dexamethasone (Selleckchem, USA) was administered to the mice by intraperitoneal injection to induce thymus apoptosis. The thymus was collected and dissociated into a single-cell suspension as previously described in refs. ^4,23^. The thymocytes were analyzed by annexin V and propidium iodide staining and flow cytometry at the Flow Cytometry Core Facility (National Cancer Center, Korea) using FACSVerse (BD Bioscience, USA) as described previously^24^.

### Chemical treatment and Western blotting

Each chemical treatment was administered 4 h prior to protein extraction. Cells were harvested and washed in PBS and then lysed in a buffer containing 20 mM Tris-HCl (pH 7.4), 150 mM NaCl, 1% (v/v) Triton X-100, 1 mM EDTA, a protease inhibitor cocktail (genDEPOT, USA), and a phosphatase inhibitor cocktail (genDEPOT, USA). Western blotting was performed according to a modified version of a previously described method^25^. After SDS–PAGE and protein transfer, the desired protein was detected using an iBind™ Automated Western system (Thermo Fisher Scientific, USA). Equal concentrations of UNC2250 were used as a positive control. Full immunoblots are shown in Supplementary Fig. 2. The antibodies and chemicals used in this study are summarized in Supplementary Table 2.

### RNAi

Small interfering RNAs (siRNAs) targeting MERTK and AXL and the negative control were designed and synthesized by BIONEER (Daejeon, Korea). THP-1 macrophages were transfected with siRNA using jetPRIME transfection reagent (Polyplus, France) according to the manufacturer’s protocol. The siRNA sequences used in this study are summarized in Supplementary Table 3.

### Statistical analyses

Statistical analysis was performed as reported previously^26^. The data were presented as the means ± standard deviations, and *P* values were calculated using a Student’s *t*-test calculator (http://graphpad.com/quickcalcs/). The data are representative of at least three separate experiments.

## Results

### The X-ray cocrystal structure shows the binding mode of BMS794833 to MERTK

**Fig. 1:**
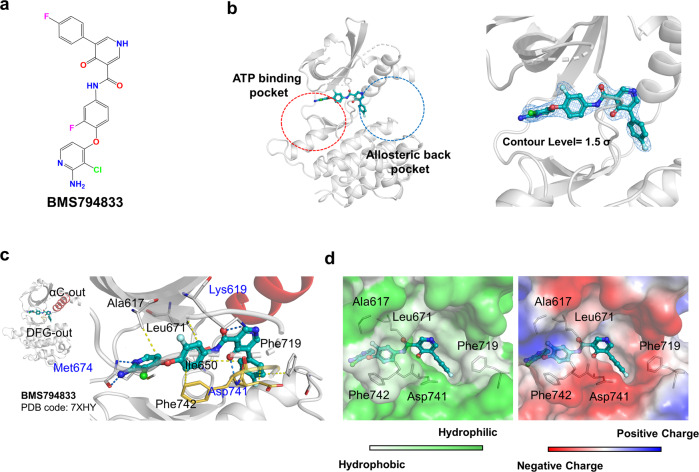
Chemical structure of BMS794833 and the X-ray crystal structure bound to MERTK. **a** The chemical structure of BMS794833. **b** BMS794833 occupies the kinase domain binding pockets from the ATP-binding pocket to the allosteric back pocket. The 2mF_o_-DF_c_ omit map is shown with a 1.5 σ contour level; BMS794833 showed good occupancy within the MERTK kinase domain. **c** The interacting hydrogen bonds between the MERTK kinase domain and BMS794833 are depicted as blue dashed lines, and hydrophobic interactions are depicted as yellow dashed lines. **d** The surface representation of the entrance region of the allosteric back pocket, also known as the hydrophobic patch. Left, the representation of hydrophobicity generated by a “color_h” script based on the previously defined hydrophobicity scale (left)^48^. Right, the representation of qualitative electrostatic potential by the “vacuum electrostatics” command in the program PyMOL.

Previously, a thermal shift assay using purified MERTK kinase domains in an in-house kinase-inhibitor library revealed that 8 of 356 compounds exhibited a melting temperature shift of >1 °C^15^. Among them, BMS794833 (IUPAC name; *N*-[4-(2-amino-3-chloropyridin-4-yl)oxy-3-fluorophenyl]-5-(4-fluorophenyl)-4-oxo-1*H*-pyridine-3-carboxamide; Fig. 1a), which is currently being studied in phase I clinical trial as a treatment for advanced solid tumors, was co-crystallized with the MERTK kinase domain. The X-ray crystal structure was determined at a resolution of 2.16 Å (Supplementary Table 1). The clear electron density map for BMS794833 showed that it occupied both the ATP-binding pocket and the allosteric back pocket (also called the hydrophobic patch) (Fig. 1b).

The main interacting forces are four hydrogen bonds (H bonds) and six hydrophobic interactions. BMS794833 formed two H bonds with the ATP-binding site and two H bonds with the allosteric back pocket of MERTK. The amine and imine at the 3-chloropyridin of BMS794833 formed H bonds with the main chain of Met674 of the ATP-binding site (Fig. 1c). The allosteric back pocket region consists of ~10 residues, of which only Lys619 and Asp741 participate in the H bond. Two oxygens in the 4-oxo-1,4-dihydropyridine-3-carboxamide group of BMS794833 formed H bonds with the side chain of Lys619 and the main chain amine of Asp741 (Fig. 1c). Three benzene ring moieties in BMS794833, namely, 3-chloropyridin-2-amine, 1-fluoro-2-methoxybenzene, and fluorobenzene, formed hydrophobic interactions with the residues of the allosteric back pocket (Fig. 1c). Specifically, 3-chloropyridine-2-amine interacts with Ala617, 1-fluoro-2-methoxybenzene interacts with Leu671 (gatekeeper) and Phe742, and fluorobenzene interacts with Ile650, Asp741, and Phe719. The solvent-exposed gate of the hydrophobic patch was mostly negatively charged and hydrophilic, while the inside of the hydrophobic patch was neutral and hydrophobic (Fig. 1d).

It is noteworthy that the H bond and hydrophobic interactions between BMS794833 and Asp741 lock the inactive conformation of the DFG motif by flipping the side chain of Asp741 out of the ATP-binding site of MERTK (Fig. 1c). No salt bridge was formed between Lys619 at the β3 strand and Glu637 at the αC, which is essential for the active conformation^10,12^, and the αC-helix extended outward from the ATP-binding site (Fig. 1c). These results show that BMS794833 binds to the inactive form of MERTK as a type II inhibitor.

### Comparison of published complex crystal structures of inhibitors and MERTK

The MERTK:BMS794833 complex was compared to 31 other MERTK:inhibitor complex structures deposited in the Protein Data Bank (PDB). Most MERTK inhibitors were type I1/2 (24 of 31), and some were type II (4 of 31). Some inhibitors (3 of 31) could not be determined because of a missing αC-helix structure (Supplementary Table 4).

**Fig. 2:**
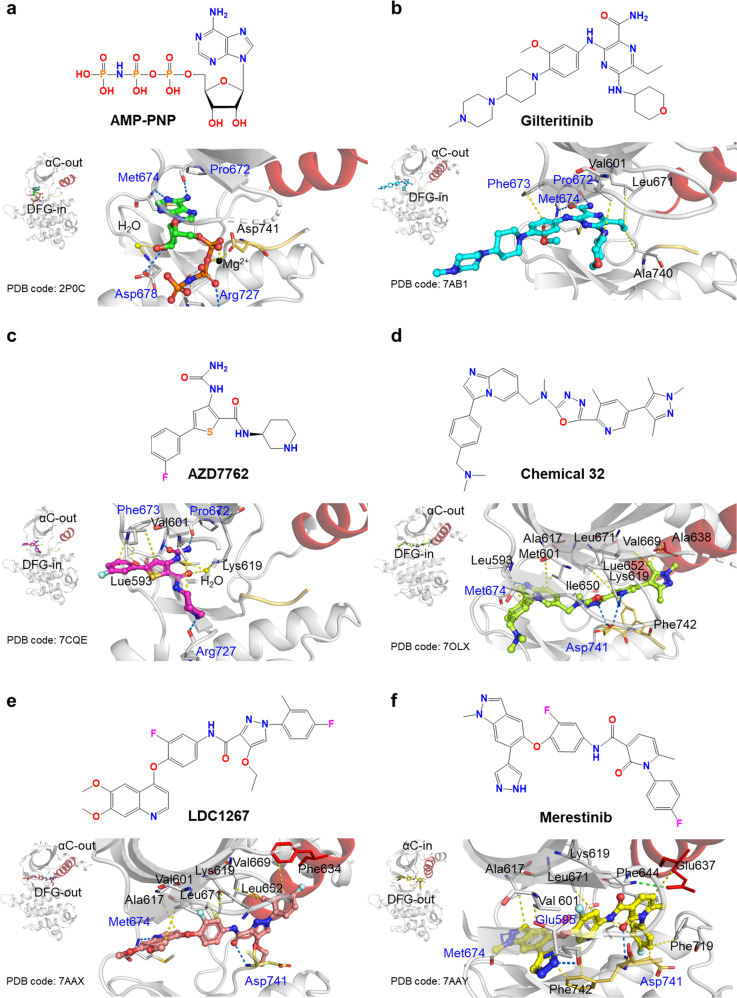
Comparison of published complex crystal structures of inhibitors and MERTK. The chemical structures of ligand molecules bound to MERTK are illustrated at the top, and key residues for ligand interaction are shown at the bottom. The conformations of the DFG motif and αC are also shown on the left. The ligands are **a** AMP-PNP, **b** gilteritinib, **c** AZD7762, **d** chemical 32, **e** LDC1267, and **f** merestinib. Hydrogen bonds are shown as blue dashed lines. Hydrophobic and water- and metal-mediated polar interactions between ligands and interacting residues are also shown in yellow.

All of the MERTK inhibitors that bind only to the ATP pocket were classified as type I1/2 with DFG-in and αC-out conformations; we found no typical type I inhibitors with DFG-in and αC-in conformations in the PDB. MERTK bound to AMP-PNP (an ATP analog compound) has a DFG-in conformation, but a salt bridge between the side chain of Glu637 in αC and the side chain of Lys619 in the β3 strand was impaired, resulting in an αC-out conformation and consequent inactivity (Fig. 2a). Of the inhibitors that bind only to the ATP pocket (16 out of 31), only gilteritinib (PDB code 7AB1) and AZD7762 (PDB code 7CQE) have entered clinical trials, and MERTK structures in complexes with these two inhibitors are very similar to the AMP-PNP-bound MERTK structure (Fig. 2b, c).

Considering that the ATP pockets of many kinases are similar, binding to additional pockets to increase specificity would be advantageous. Some type I1/2 inhibitor structures deposited in the PDB show that a series of MERTK inhibitors occupy the ATP and allosteric back pockets of MERTK (8 out of 31). For example, Chemical 32 (PDB code 7OLX; 1,3,4-oxadiazole, pyridine, 1,3,5-trimethyl-1H-pyrazole) developed by AstraZeneca occupies the allosteric back pocket; imines at 1,3,4-oxadiazole and pyridine form strong H bonds with Asp741 to establish the active form with the DFG-in conformation. In addition, the methyl group of 1,3,5-trimethyl-1H-pyrazole forms a hydrophobic interaction with Ala638, displacing the αC-out (Fig. 2d).

All type II inhibitors (4 out of 31) deposited in the PDB simultaneously bind to the ATP pocket and the allosteric back pocket of MERTK. Three inhibitors can be classified as typical type II, with DFG-out and αC-out conformations. For example, in LDC1267 (PDB code 7AAX), the DFG motif in the inactive form showed the DFG-out position, DFG-Phe was turned inward to the ATP-binding site, and DFG-Asp was flipped out to the allosteric back pocket (Fig. 2e). Merestinib (PDB code 7AAY) has a unique feature in which DFG-out and αC-in form a canonical salt bridge (Lys619 and Glu637) (Fig. 2f). 1,6-Dimethylpyridin-2(1H)-one forms a hydrophobic interaction with Glu637 at αC, turning the αC-helix inward to show the active conformation; however, many hydrophobic and H bond interactions between merestinib and Asp741 and Phe742 stabilize the DFG-out conformation to lock the inactive conformation.

BMS794833 is a typical type II inhibitor with a DFG-out and αC-out conformation (Fig. 1c), with a similar binding mode to MERTK as LDC1267 but similar to merestinib in its chemical structure (Figs. 1a, 2f). BMS794833 and merestinib show high similarity in the core scaffold (with a significant difference in the additive moieties of the 2-fluorophenol ring). Due to this high similarity, they show similar occupancies in the ATP and allosteric back binding pockets, but the interacting residues differ. The carbonyl group in an acetamide is the same for BMS794833 and merestinib; however, BMS794833 forms an H bond with Lys619, hindering the formation of a salt bridge with αC, while merestinib does not.

### BMS794833 inhibits MERTK kinase activity in vitro and in cells

**Fig. 3:**
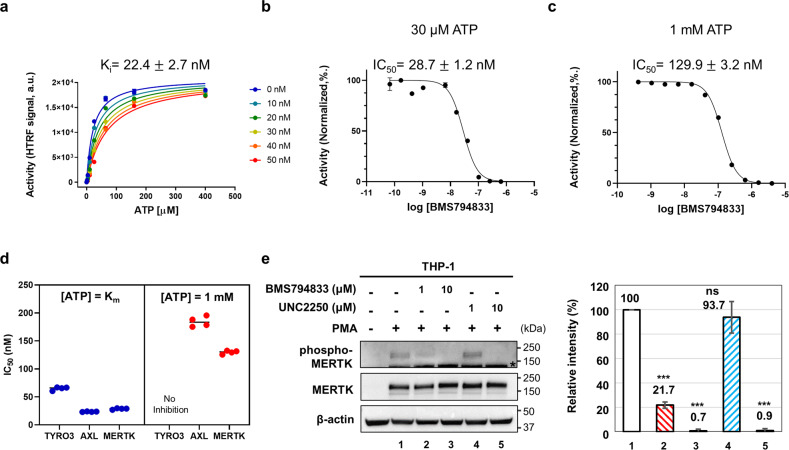
BMS794833 inhibits MERTK kinase activity both in vitro and in cells. **a** Michaelis–Menten curve for MERTK kinase activity at different BMS794833 concentrations. **b**, **c** IC_50_ values of BMS794833 at ATP concentrations near its *K*_m_ (30 μM) and near the intracellular level (1 mM). **d** The IC_50_ of BMS794833 in the TAM family. **e** THP-1 monocytes were differentiated by treatment with 100 nM PMA for 2 days. The indicated concentrations of BMS794833 and UNC2250 were administered 4 h prior to cell harvesting, and the expression levels of MERTK and phosphorylated MERTK (Y749, Y753, and Y754) were evaluated by Western blotting. β-Actin was used as a loading control. *Nonspecific bands. Relative intensities of phosphorylated MERTK bands normalized to MERTK bands are shown on the right. The values are presented as the mean ± SD from three independent experiments. ***P < 0.001; ns not significant.

Based on the clear binding mode of MERTK and BMS794833, we performed HTRF kinetic assays to ascertain how BMS794833 inhibits MERTK. We found that BMS794833 inhibits MERTK in an ATP competitive inhibition mode with a *K*_i_ value of 22.4 nM (Fig. 3a and Table 1). The IC_50_ values for the inhibitor at ATP concentrations near *K*_m_ (30 μM)^15^ and intracellular ATP levels (1 mM) were 28.7 nM (±1.2 nM) and 129.9 nM (±3.2 nM), respectively (Fig. 3b, c). BMS794833 also inhibited all TAM family proteins at low ATP concentrations but inhibited MERTK most effectively at intracellular ATP concentrations (Fig. 3d). Next, we investigated whether BMS79488 inhibited MERTK activity in cells. The human monocyte cell line THP-1 was differentiated into macrophages by phorbol-12-myristate 13-acetate (PMA) treatment, and MERTK was expressed only under PMA treatment (Fig. 3e). The intracellular activity of MERTK can be monitored through autophosphorylation of the Y749, Y753, and Y754 residues^27^. In Western blotting analyses, the MERTK phosphorylation band of THP-1 macrophages was significantly reduced by treatment with 1 μM BMS794833, whereas UNC2250, a selective MERTK inhibitor^28^, inhibited MERTK at 10 μM (Fig. 3e).

**Table 1:** Michaelis–Menten kinetics of MERTK at different BMS794833 concentrations.

BMS794833	0 nM	10 nM	20 nM	30 nM	40 nM	50 nM
*V*_max_	20,805 ± 488	20,871 ± 176	20,242 ± 178	20,948 ± 265	21,462 ± 141	21,639 ± 361
*K*_m_ (μM)	23.52 ± 0.54	26.88 ± 0.63	36.98 ± 1.30	54.52 ± 0.30	65.86 ± 0.82	79.45 + 3.21

### Real-time efferocytosis assay using human monocyte cell lines

**Fig. 4:**
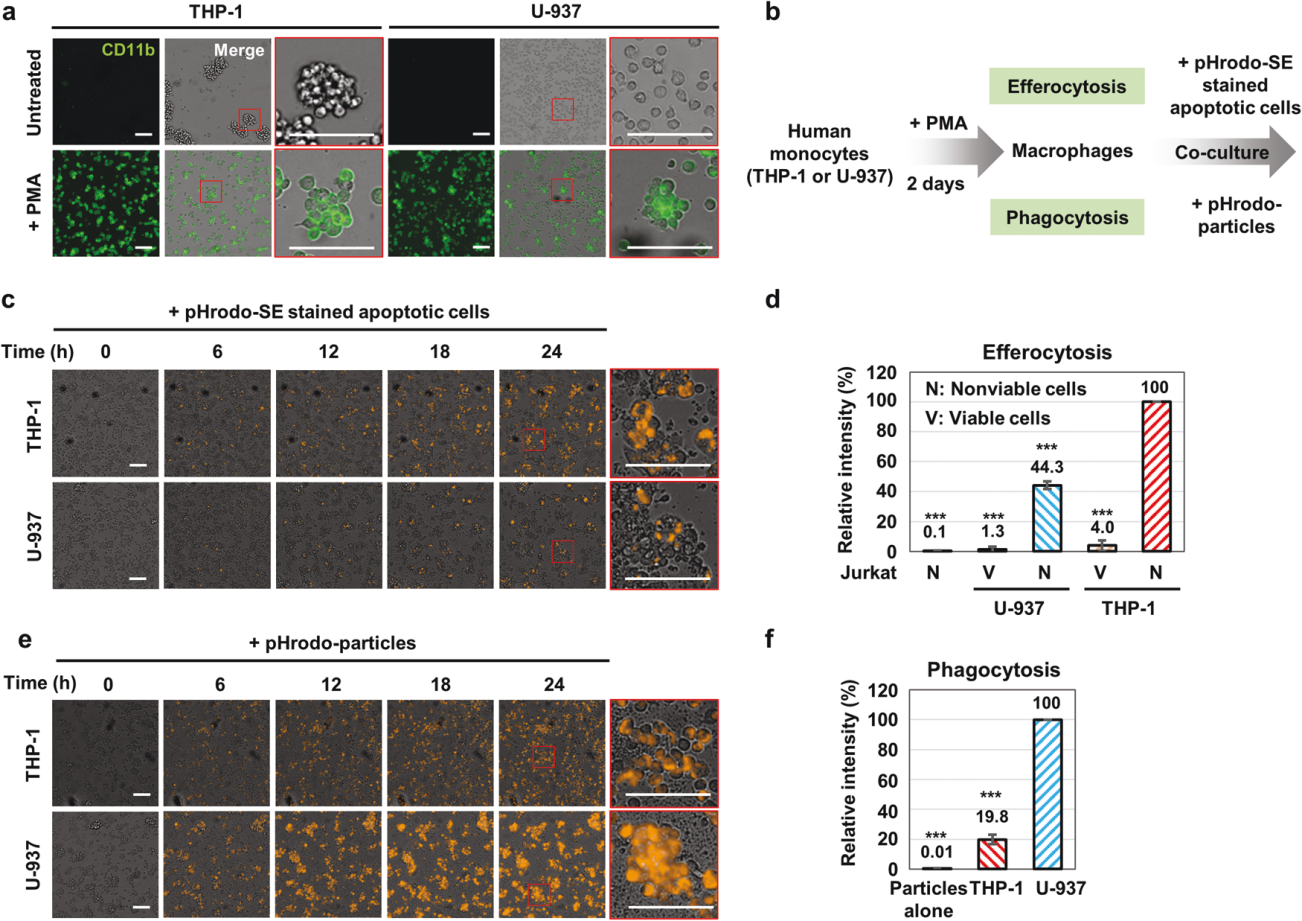
Real-time efferocytosis monitoring system with human-derived monocytic cell lines. **a** CD11b expression in THP-1 and U-937 cells was investigated by immunocytochemistry. Cells were treated with 100 nM PMA (or DMSO) for 48 h. **b** Schematic representation of efferocytosis and phagocytosis assays. **c**, **d** Time-lapse microscopy and relative intensity of THP-1 and U-937 macrophages after coculture with nonviable Jurkat cells stained with pHrodo-SE for efferocytosis or pHrodo-particles for phagocytosis **e**, **f**. Merged images were obtained at 6 h intervals starting at 0 h of the coculture. Each zoomed-in view of the selected area is marked in red. The values are presented as the mean ± SD from at least three independent experiments. ***P < 0.001. Scale bar: 100 μm.

Next, we determined whether BMS794833 inhibited the activity of MERTK sufficiently to affect the physiological outcomes of cells. MERTK inhibition must inhibit efferocytosis in macrophages to have an anticancer effect^7^. To this end, we established real-time efferocytosis assays using the THP-1 and U-937 human monocyte cell lines. Monocytes proliferate in suspension culture, but they lose proliferation ability and become adherent when differentiated into macrophages^29^. Immunofluorescent staining with an antibody specific for CD11b, known as a macrophage marker^29^, showed that most THP-1 and U-937 cells were differentiated into macrophages when 100 nM PMA was administered for 48 h (Fig. 4a).

We investigated whether monocyte-derived macrophages exhibit efferocytosis and phagocytosis. Apoptotic Jurkat cells stained with pHrodo-SE were added to macrophages to test for efferocytosis (Fig. 4b). Exposure of PtdSer to the surface of apoptotic cells induces macrophage efferocytosis^30^. Because the pHrodo™ Red dye fluoresces only in an acidic environment, it appears red only when swallowed by macrophages. Cisplatin treatment was used to induce apoptosis in Jurkat cells to generate apoptotic cells for efferocytosis assays^31,32^. Flow cytometry analysis after Annexin V and propidium iodide staining showed that cisplatin treatment at 50 μM for 2 days resulted in ~95% nonviable cells, and most of these were positive for Annexin V (Supplementary Fig. 1a, b). After pHrodo-SE staining and coculture with THP-1 and U-937 monocyte-derived macrophages, red fluorescence increased over time when apoptotic cells were added but not when viable cells were added (Fig. 4c, Supplementary Fig. 1c, and Supplementary Movies 1–5). The red fluorescence intensity was saturated at 24 h. Nonviable Jurkat cells stained with pHrodo-SE alone did not show red fluorescence. These results demonstrate that the extent of efferocytosis over time can be monitored effectively using THP-1 and U-937 monocyte-derived macrophages. In addition, because efferocytosis occurs more frequently in THP-1 cells than in U-937 cells (Fig. 4d), THP-1 cells appear to be more suitable for observing the extent of efferocytosis.

For the phagocytosis analysis, pHrodo-particles were added to macrophages (Fig. 4b). The gram-positive commensal *S*. *aureus* bacterium induces macrophage recruitment, polarization, and phagocytosis^33^. THP-1 and U-937 monocyte-derived macrophages to which we added pHrodo-particles showed an increase in red fluorescence over time, whereas PHrodo-particles alone did not (Fig. 4e, Supplementary Fig. 1d, and Supplementary Movies 6–8). Unlike efferocytosis, phagocytosis occurred more frequently in U-937 cells than in THP-1 cells (Fig. 4f).

### Efferocytosis of THP-1 macrophages depends on MERTK activity

**Fig. 5:**
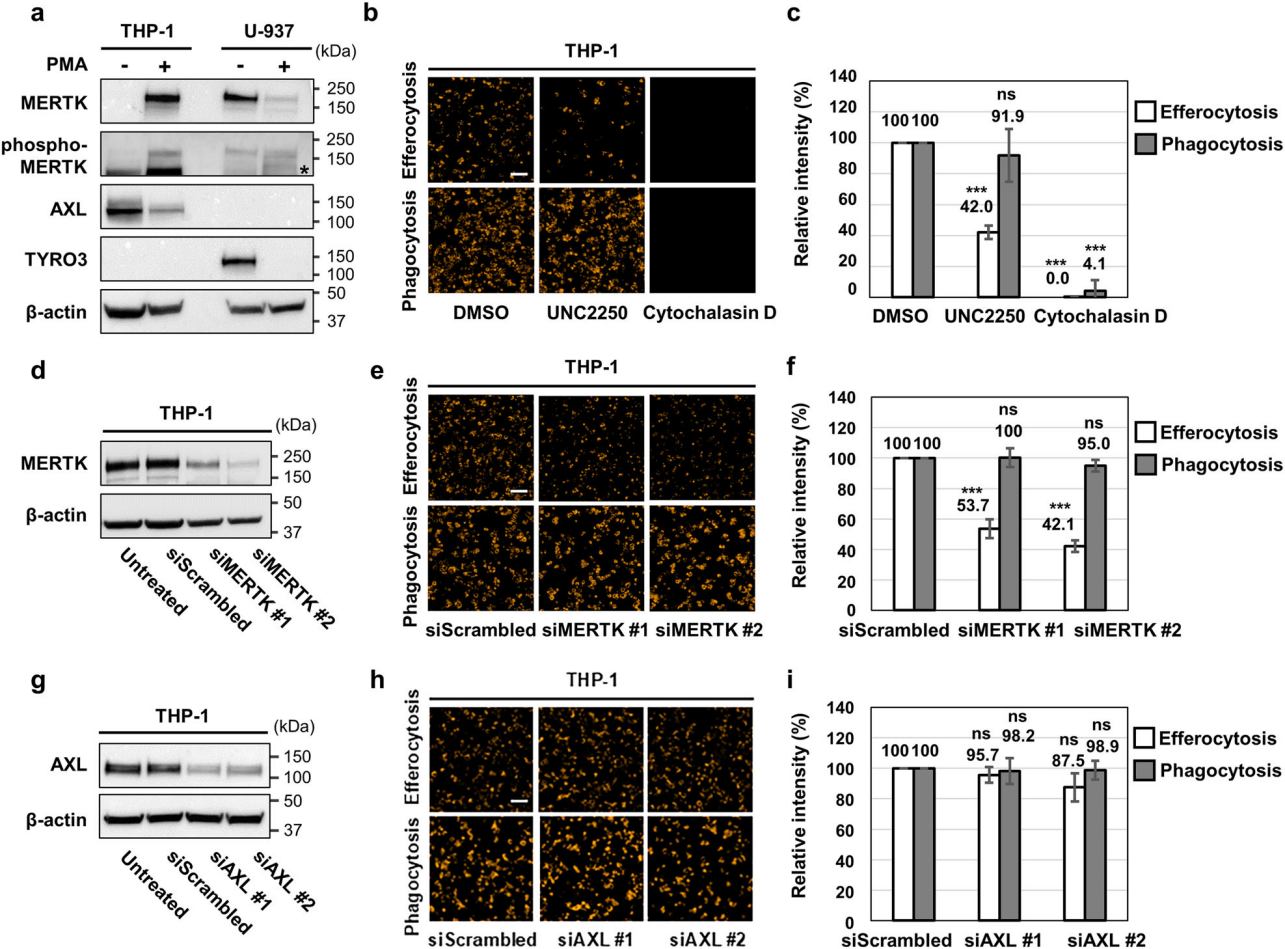
Efferocytosis of THP-1 macrophages depends on MERTK activity. **a** Western blotting analysis of THP-1 and U-937 cells to measure the expression levels of the TAM family and MERTK active form (phospho-MERTK). Cells were treated with 100 nM PMA (or DMSO). **b**, **c** Images and relative intensity in efferocytosis and phagocytosis assays. Before coculture, the cells were pretreated with 10 μM UNC2250 or cytochalasin D for 1 h. All of the data were obtained after 24 h of coculture. **d** The knockdown efficiency of siMERTKs in THP-1 cells was measured via Western blotting. Each siRNA was applied at a final concentration of 50 nM. **e**, **f** Images and relative intensity in efferocytosis and phagocytosis assays. Before coculture, siMERTKs were pretreated with 50 nM for 24 h. **g** The knockdown efficiency of siAXLs in THP-1 cells was measured via Western blotting analysis. **h**, **i** Images and relative intensity in efferocytosis and phagocytosis assays. The values are presented as the mean ± SD from at least three independent experiments. ***P < 0.001; ns not significant; Scale bar: 100 μm.

As we established real-time monitoring systems for efferocytosis and phagocytosis, we determined whether these phenomena depended on MERTK activity. First, the Western blotting analysis showed that MERTK was not expressed in THP-1 monocytes but was expressed in differentiated macrophages. Detection of MERTK autophosphorylation suggested that MERTK was activated in THP-1 monocyte-derived macrophages (Fig. 5a). However, the expression of AXL decreased with the differentiation of THP-1 monocytes, and TYRO3 expression was not detected (Fig. 5a), suggesting that MERTK has a pivotal role among the TAM family members in THP-1 monocyte-derived macrophages. In U-937 cells, MERTK and TYRO3 were expressed in monocytes, but their expression decreased after differentiation, and AXL was not detected (Fig. 5a).

Next, we investigated whether the inhibition of MERTK affects the efferocytosis of THP-1 monocyte-derived macrophages. The addition of UNC2250, a selective MERTK inhibitor^28^, significantly reduced the efferocytosis but not phagocytosis of THP-1 macrophages. As a control, the addition of cytochalasin D, which inhibits actin reorganization by depolymerizing actin filaments^34^, completely inhibited both efferocytosis and phagocytosis (Fig. 5b, c). Knockdown of MERTK by siRNA confirmed by Western blotting (Fig. 5d) reduced the efferocytosis but not phagocytosis of THP-1 monocyte-derived macrophages (Fig. 5e, f). In contrast, knockdown of AXL (Fig. 5g) did not affect efferocytosis or phagocytosis (Fig. 5h, i). These results suggest that the real-time efferocytosis monitoring system of THP-1 macrophages can be used to test MERTK inhibitor efficacy, as the efferocytosis of THP-1 monocyte-derived macrophages depends on MERTK activity.

### BMS794833 inhibits efferocytosis at the cellular and animal levels

**Fig. 6:**
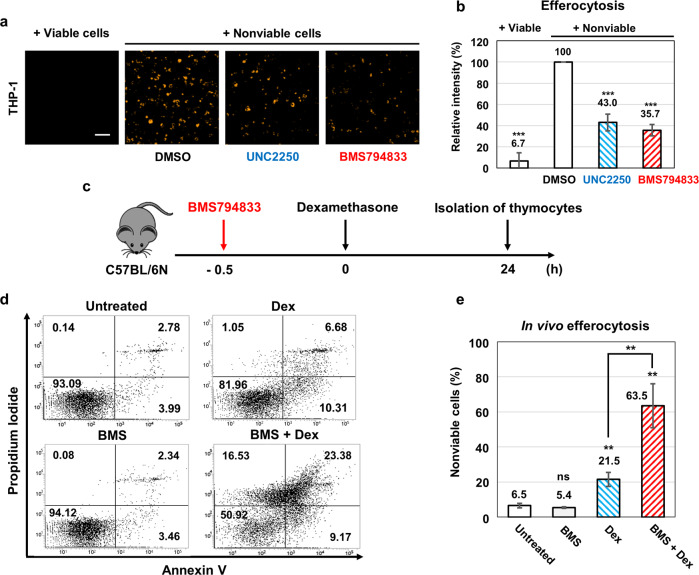
BMS794833 inhibits efferocytosis at the cellular and animal levels. **a**, **b** Images and relative intensity in efferocytosis assays with 10 µM UNC2250 or BMS794833. Cells were pretreated with the inhibitors 1 h before coculture. All images and data were obtained after 24 h of coculture. **c** Schematic depiction of the in vivo efferocytosis assay. Mice were dosed (or not) with BMS794833 30 min before dexamethasone treatment. Each thymus was collected and isolated as thymocytes 24 h after dexamethasone treatment. **d**, **e** Results of in vivo efferocytosis. Thymocytes were stained with Annexin V and propidium iodide. The values are presented as the mean ± SD from at least three independent experiments. **P < 0.01, ***P < 0.001; ns not significant; scale bar: 100 μm; Dex dexamethasone, BMS BMS794833.

We investigated whether BMS794833 could inhibit the efferocytosis of macrophages. Treatment with 10 μM BMS794833 significantly decreased the efferocytosis of THP-1 monocyte-derived macrophages (Fig. 6a, b). UNC2250, which has an IC_50_ of 1.7 nM for MERTK in cell-free assays^28^, showed a slightly weaker efferocytosis inhibitory effect than BMS794833 at 10 μM (Fig. 6a, b), which proved that BMS794833 is a sufficiently potent inhibitor of MERTK.

Because BMS794833 has entered clinical trials and is safe for animals, we tested whether it could inhibit efferocytosis in animals. MERTK kinase inhibitors are expected to act similarly in humans and mice, as their respective MERTK kinase domains exhibit high structural similarity and are 94% similar in sequence. An in vivo efferocytosis assay using C57BL/6 N immunocompetent mice was performed by observing the clearance of apoptotic thymocytes induced by dexamethasone treatment (Fig. 6c). In a previous study, intraperitoneal injection of dexamethasone caused an accumulation of nonviable cells in the thymi of mice 8 h after treatment, which was cleared by thymic resident macrophages 24 h after treatment^7^. A single dexamethasone treatment somewhat increased the number of apoptotic thymocytes 24 h after treatment, suggesting that considerable numbers of apoptotic thymocytes were cleared by efferocytosis. Pretreatment with BMS794833 before dexamethasone treatment significantly increased the percentage of apoptotic thymocytes (Fig. 6d, e), suggesting that BMS794833 inhibits efferocytosis in the thymus. As a control, treatment with BMS794833 alone did not affect the percentage of apoptotic thymocytes (Fig. 6d, e). Overall, these data show that BMS794833 is a type II inhibitor of MERTK and inhibits efferocytosis of macrophages in animals. It has shown great potential for further development as a clinically applicable MERTK inhibitor.

## Discussion

Because of the significant potential of MERTK as an anticancer target, many MERTK inhibitors are under development. Three compounds whose X-ray cocrystal structures with MERTK have been revealed have entered clinical trials: AZD7762, gilteritinib, and merestinib. However, further development of AZD7762 was halted due to unpredictable cardiac toxicity^35^. Gilteritinib, whose primary target is FLT3, has been approved by the Food and Drug Administration (FDA) for the treatment of acute myeloid leukemia^14,36^. This drug also inhibits several other kinases, such as ALK, LTK, AXL, and MERTK. At 1 nM, it inhibits FLT3 by 86.8%, ALK by 76.1%, LTK by 81.8%, AXL by 54.3%, and MERTK by 21.5%, which indicates a relatively weak inhibitory effect on MERTK^37^. Merestinib has passed phase I clinical trials and is now in phase II clinical trials for the treatment of advanced or metastatic biliary tract cancer^38^. Its primary target is MET, but it also inhibits MST1R, FLT3, AXL, PDGFRA, ROS1, TEK, DDR1/2, and MKNK-1/2^16^. This drug is a type II inhibitor of MERTK with DFG-out and αC-in conformations. BMS794833, identified in this study, has entered phase I clinical trials for the treatment of advanced or metastatic solid tumors (ClinicalTrials.gov Identifier: NCT00792558). Although the primary targets of BMS794833 are MET and VEGFR2^39^, BMS794833 also inhibits the polarization of monocytes into tumor-associated macrophages through a polypharmacological effect rather than inhibition of MET and VEGFR2^40^. BMS794833 is a typical type II inhibitor of MERTK with DFG-out and αC-out structural conformations and inhibits efferocytosis in cells and animal models. Thus, this compound is a promising candidate anticancer agent that inhibits MERTK.

One of the biggest obstacles to developing MERTK inhibitors is the lack of suitable and efficient cell-based assays. Current MERTK inhibitor assays can be roughly divided into three categories. One class measures autophosphorylated MERTK via Western blotting or enzyme-linked immunosorbent assay (ELISA)^41,42^. While this clearly shows whether the inhibitor inhibits MERTK, it has the disadvantage of not showing whether the inhibitor inhibits MERTK sufficiently to affect cell physiology. Another class measures the extent to which MERTK inhibition induced cell growth inhibition and death via proliferation assays or cell viability assays^42,43^. A disadvantage is that it is unclear whether growth inhibition and death occur in a MERTK-dependent manner. Furthermore, considering that the main role of MERTK is to modulate innate immunity rather than to regulate cell growth and death, it is unclear whether this assay adequately measures the endpoint of MERTK inhibition. The other class measures the extent to which MERTK inhibitors reduce macrophage phagocytosis or efferocytosis^9,44^. Among them, phagocytosis assays are problematic because it remains unclear whether MERTK regulates phagocytosis. In this study, we found that MERTK inhibition reduced efferocytosis but did not affect phagocytosis in THP-1 macrophages (Fig. 5). Therefore, to test the efficacy of MERTK inhibitors, we should investigate efferocytosis and not phagocytosis. The extent of efferocytosis has been analyzed using either monocytes extracted from blood^9^ or monocyte cell lines such as THP-1, U-937, and RAW264.7^45–47^; however, it remains unclear whether the efferocytosis of macrophages differentiated from these monocytes is MERTK-dependent. In this study, THP-1-derived macrophages mainly expressed only MERTK among the TAM family, and efferocytosis occurred in a MERTK-dependent manner (Fig. 5). On the other hand, U-937-derived macrophages rarely expressed TAM family proteins (Fig. 5a), so efferocytosis might occur depending on factors other than the TAM family. Therefore, an efferocytosis assay using THP-1-derived macrophages might be a good method to validate MERTK inhibitors at the cellular level.

**Fig. 7:**
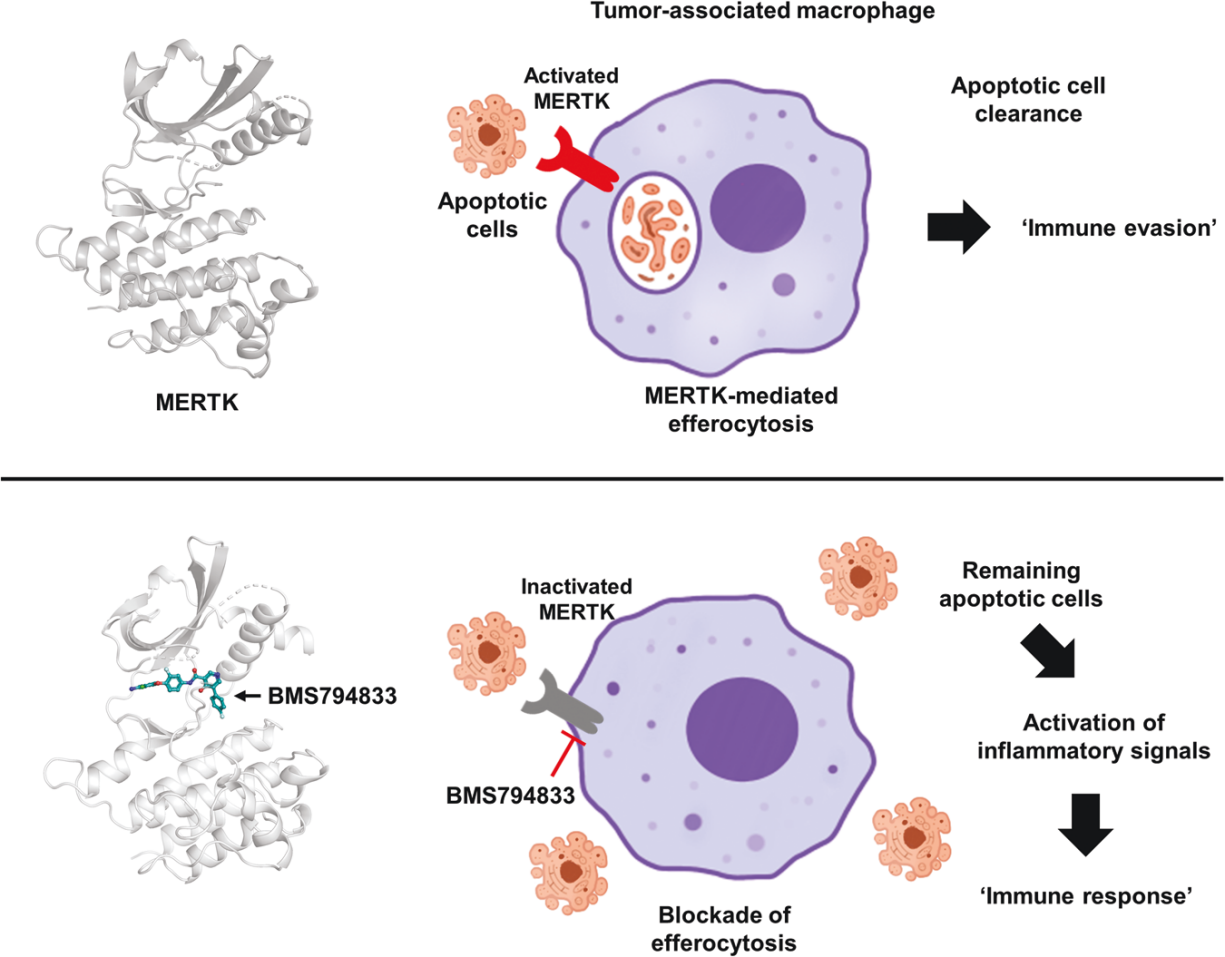
Schematic diagram suggesting BMS794833 as a cancer immunotherapy candidate. The immune response occurs via the activation of inflammatory signals induced by apoptotic cells, which are inevitably generated during cancer cell growth. Cancer cells have been known to evade the immune response by eliminating apoptotic cells through efferocytosis of macrophages. In this study, it was revealed that BMS794833 is a type II inhibitor that directly binds to MERTK and blocks efferocytosis. BMS794833 has the potential to be used as an immuno-oncology agent that prevents cancer cells from evading immunity either by itself or through modifications.

Some cells undergo apoptosis during cancer cell growth, and these cells trigger an inflammatory response to induce an immune response. One way cancer cells evade the immune response is by converting the surrounding monocytes into tumor-associated macrophages, which eliminate apoptotic cells through efferocytosis. Recently, it was reported that BMS794833 could block monocyte conversion into tumor-associated macrophages through a mechanism that is not precisely determined^40^. Because MERTK is known to affect tumor-associated macrophage polarization by modulating efferocytosis^8^ and this study revealed that BMS794833 is a type II inhibitor that directly binds to MERTK, the blockade of conversion in that study may have been caused by MERTK inhibition. In addition, BMS794833 inhibited cancer growth in immunocompetent animal models^40^. Given these facts, BMS794833 has the potential to be used as an immuno-oncology agent that prevents cancer cells from evading immunity by efferocytosis (Fig. 7).

Overall, this study shows that BMS794833 is a potent and novel MERTK inhibitor that modulates efferocytosis. Efferocytosis occurs in a MERTK-dependent manner in THP-1-derived macrophages, suggesting the possibility of using this assay as a fluorescent-based high-throughput screening process to identify MERTK inhibitors.

## Supplementary information

Supplementary Information

Supplementary Movie 1

Supplementary Movie 2

Supplementary Movie 3

Supplementary Movie 4

Supplementary Movie 5

Supplementary Movie 6

Supplementary Movie 7

Supplementary Movie 8
