# Author Correction: BMS794833 inhibits macrophage efferocytosis by directly binding to MERTK and inhibiting its activity

**DOI:** 10.1038/s12276-026-01638-x

**Published:** 2026-01-14

**Authors:** Seung-Hyun Bae, Jung-Hoon Kim, Tae Hyun Park, Kyeong Lee, Byung Il Lee, Hyonchol Jang

**Affiliations:** 1 Reasearch Institute, National Cancer Center, Goyang, 10408 Republic of Korea; 2 Department of Cancer Biomedical Science, National Cancer Center Graduate School of Cancer Science and Policy, Goyang, 10408 Republic of Korea; 3 Department of Anesthesiology, Weill Cornell Medical College, New York, NY 10065 USA; 4 College of Pharmacy, Dongguk University-Seoul, Goyang, 10326 Republic of Korea

Correction to: *Experimental & Molecular Medicine* 10.1038/s12276-022-00840-x, published online 02 September 2022

After online publication of this article, the authors noticed an error in the Fig. 5h section.

During figure assembly, the image corresponding to the siAXL#1 condition in Fig. 5h was inadvertently replaced with the siScrambled image from Fig. 5e.
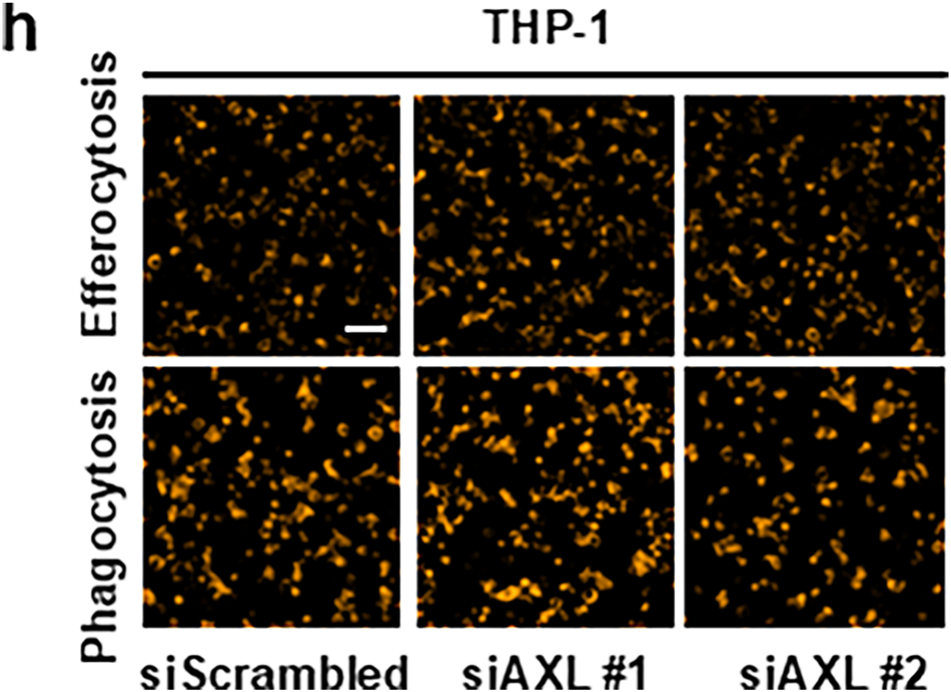


The correct siAXL#1 image was obtained from the same experiment performed under identical conditions. Original data are available upon request.

This error does not affect the quantitative analyses, statistical results, or the conclusions of the manuscript. The authors apologize for any inconvenience caused.

The original article has been corrected.

