# Supplementary material for: BMS794833 inhibits macrophage efferocytosis by directly binding to MERTK and inhibiting its activity
Source: Exp Mol Med. 2022 Sep 2;54(9):1450–60. doi: 10.1038/s12276-022-00840-x (PMC9534909; doi:10.1038/s12276-022-00840-x)
Supplement: Supplementary file 1 — Supplementary Information [file 12276_2022_840_MOESM1_ESM.pdf]

Supplementary Information

BMS794833 inhibits macrophage efferocytosis by directly binding to MERTK and inhibiting its activity

List of Supplementary Information

Supplementary Fig. 1. (Related to Fig. 4) Time-lapse images of efferocytosis and phagocytosis

Supplementary Fig. 2. Full immunoblots

Supplementary Table 1. Data collection and refinement statistics

Supplementary Table 2. Antibodies and chemicals used in this study

Supplementary Table 3. siRNA sequences

Supplementary Table 4. MERTK: inhibitor complex structures deposited in the Protein Data Bank (PDB)

Supplementary Movie 1 (Related to Fig. 4c). Time-lapse microscopy of pHrodo-SE stained nonviable Jurkat alone shown 1-h intervals for 24 h

Supplementary Movie 2 (Related to Fig. 4c). Time-lapse microscopy of THP-1 macrophages with pHrodo-SE stained viable Jurkat

Supplementary Movie 3 (Related to Fig. 4c). Time-lapse microscopy of THP-1 macrophages with pHrodo-SE stained nonviable Jurkat

Supplementary Movie 4 (Related to Fig. 4c). Time-lapse microscopy of U-937 macrophages with pHrodo-SE stained viable Jurkat

Supplementary Movie 5 (Related to Fig. 4c). Time-lapse microscopy of U-937 macrophages with pHrodo-SE stained nonviable Jurkat

Supplementary Movie 6 (Related to Fig. 4e). Time-lapse microscopy of pHrodo-particles alone

Supplementary Movie 7 (Related to Fig. 4e). Time-lapse microscopy of THP-1 macrophages with pHrodo-particles

Supplementary Movie 8 (Related to Fig. 4e). Time-lapse microscopy of U-937 macrophages with pHrodo-particles

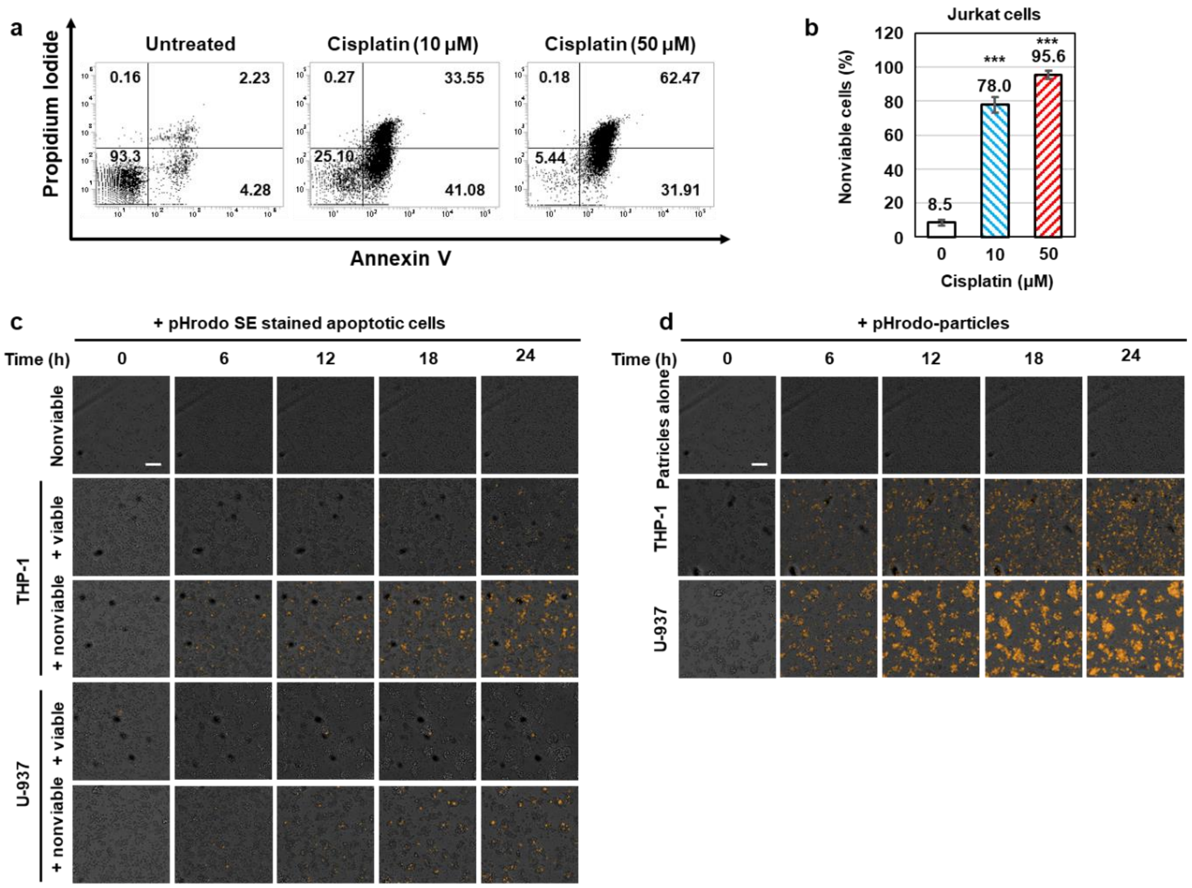

Supplementary Fig. 1. (Related to Fig. 4) **Time-lapse images of efferocytosis and phagocytosis**

(a and b) Jurkat cells were treated with cisplatin for 48 h and stained with Annexin V and propidium iodide. Flow cytometric analysis was performed to estimate the percentage of nonviable cells (mean ± SD; n = 3). (c) Time-lapse microscopy of THP-1 and U-937 after coculture with nonviable (or viable) Jurkat cells stained with pHrodo-SE (50 ng/mL). Merged (bright field and efferocytosis signals) images were obtained at 6 h intervals starting at 0 h of the coculture. (d) Time-lapse microscopy of THP-1 and U-937 after coculture with pHrodo-particles for phagocytosis. Merged (bright-field and phagocytosis signals) images were shown.

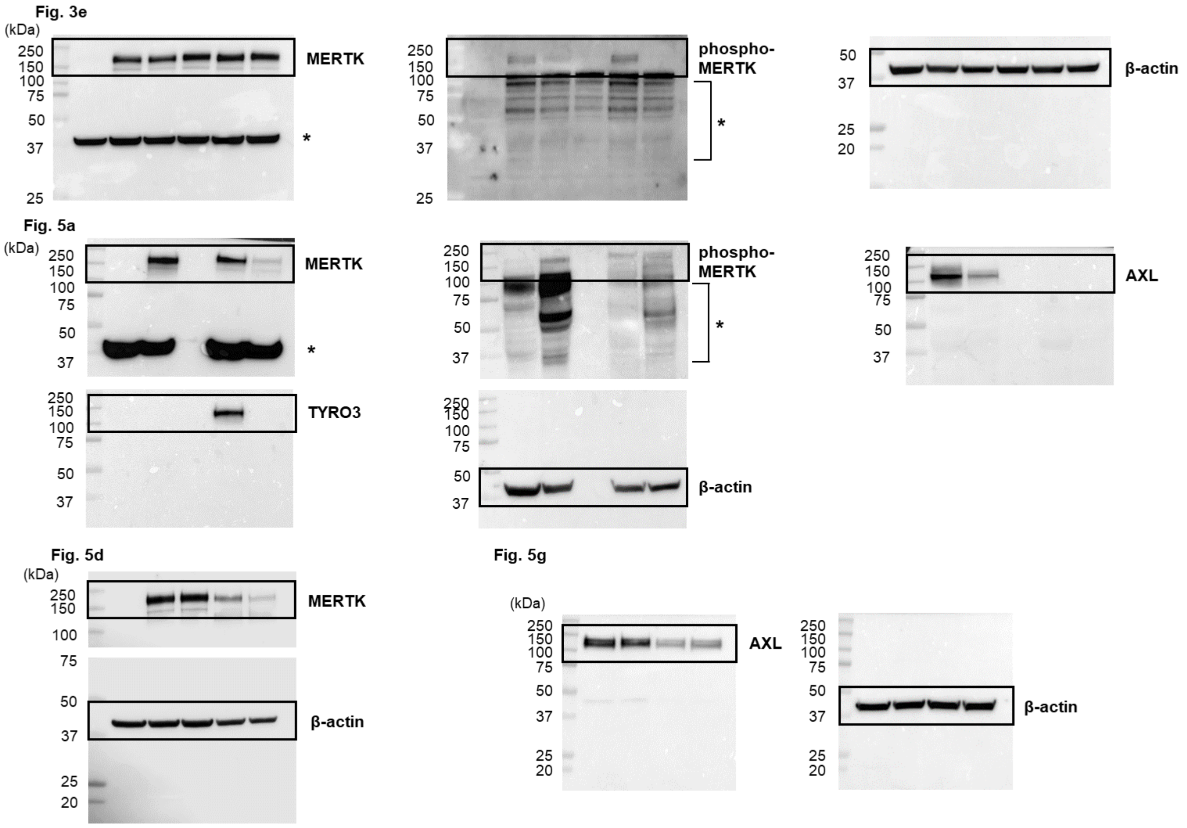

Supplementary Fig. 2. Full immunoblots. Full-length images of Western blotting analyses.
*Nonspecific bands

Supplementary Table 1. Data collection and refinement statistics

Dataset	MERTK kinase domain: BMS794833
**Diffraction data statistics**	
X-ray source	PLS-11C
Wavelength (Å)	0.979
Space group	$C222_1$
Cell parameters	
a, b, c (Å)	92.127, 92.533, 71.435
α, β, γ (°)	90, 90, 90
Resolution range (Å)	50–2.16 (2.20–2.16)*
R_{sym} (%)	8.1 (47.8)
$R_{p.i.m.}$ (%)	3.4 (21.8)
$CC_{1/2}$	0.985 (0.931)
Mean I/σI	23.2 (2.1)
Redundancy	6.8 (4.7)
Completeness (%)	98.5 (80.5)
No. of unique reflections	16,509 (657)
**Refinement statistics**	
Resolution range (Å)	38.71–2.16
R_{work}/R_{free} (%)	20.8/25.2
No. of nonhydrogen atoms/average B-factor (Å^2)	
Protein (MERTK kinase domain)	2,167/47.73
Solvent	40/41.23
Chloride	4/50.29
Dimethyl sulfoxide	4/52.61
Ligand (BMS794833)	33/36.53
RMS deviation	
Bond length (Å)	0.008
Bond angles (°)	1.03
Ramachandran plot (%)	
Favored/outliers	96.96/0
Clash score	5.47
PDB entry	7XHY

*Values in parentheses are for the highest-resolution shell.

Supplementary Table 2. Antibodies and chemicals used in this study

Name	Manufacturer	Catalog no.
Anti-phospho MERTK (Tyr749/753/754)	PhosphoSolutions	p186-749
Anti-MERTK	Abcam	ab52968
Anti-AXL	Cell Signaling	8661S
Anti-TYRO3	Cell Signaling	5585S
Anti-β-actin	Sigma-Aldrich	A2228
Goat anti-mouse IgG, HRP-conjugated	Pierce	31430
Goat anti-rabbit IgG, HRP-conjugated	Pierce	31460
Alexa Fluor® 647 anti-mouse/human CD11b	BioLegend	101218
Phorbol 12-myristate 13-acetate (PMA)	Sigma-Aldrich	P8139
cis-Diammineplatinum (II) dichloride (cisplatin)	Sigma-Aldrich	P4394
pHrodo™ Red, succinimidyl ester	Thermo Fisher Scientific	P35372
pHrodo™ Red S. aureus Bioparticles™	Thermo Fisher Scientific	A10010
Dexamethasone Sodium Phosphate	SelleckChem	S4028
UNC2250	SelleckChem	S7342
AZD7762	SelleckChem	S1532
BMS794833	Cayman Chemical Company	26180
Cytochalasin D	Cayman Chemical Company	11330
Kolliphor	Sigma-Aldrich	C5135

Supplementary Table 3. siRNA sequences

siRNA	Sequences*	Cat No.
siMERTK #1	5’ CUCAUGAAGGACGGUACAUtt 3’	10461-1
	3’ ttGAGUACUUCCUGCCAUGUA 5’	
siMERTK #2	5’ AGAUGACAUGACUGUCUGUtt 3’	10461-2
	3’ ttUCUACUGUACUGACAGACA 5’	
siAXL #1	5’ GACUGUCUGGAUGGACUGUtt 3’	558-1
	3’ ttCUGACAGACCUACCUGACA 5’	
siAXL #2	5’ GACUCUAGAGUCCAAGGUUtt 3’	558-3
	3’ ttCUGAGAUCUCAGGUUCCAA 5’	
siScrambled	Not provided	SS-1011

*Target sequences are marked with capital letters.

Supplementary Table 4. MERTK: inhibitor complex structures deposited in the Protein Data Bank (PDB)

Types	Type I1/2		Type II		N.D.**
Conformation	DFG-in αC-out		DFG-out αC-out	DFG-out αC-in	N.D.
Binding site	ATP pocket	ATP pocket + allosteric	ATP pocket + allosteric		ATP pocket
PDB code* (name)	2P0C (AMP-PNP)***	6MEP (UNC3437)	7AAX (LDC1267)	7AAY (merestinib)	4M3Q (UNC1917)
	3BPR	7AAZ	7AW1		5TC0
	3BRB (ADP)***	7AVX (NPS1034)	7AW4		5TD2
	3TCP (UNC569)	7AVY			
	4MH7 (UNC1896)	7AW2			
	4MHA (UNC1817)	7OLS			
	5K0K (UNC2434)	7OLV			
	5K0X (UNC2541)	7OLX (chemical 32)			
	5U6C				
	7AB1 (gilteritinib)				
	7AB2 (UNC2025)				
	7AVZ				
	7AW0				
	7AW3				
	7CQE (AZD7762)				
	7DXL				
	7M5Z				
	7OAM				

*Last update: April 30, 2022

**N.D.: Not determined

***Ligand and ligand analog (not inhibitors)
